# Terms, definitions and measurements to describe the sonographic features of adnexal tumors: updated consensus opinion from the International Ovarian Tumor Analysis (IOTA) Group

**DOI:** 10.1002/uog.70191

**Published:** 2026-04-07

**Authors:** D. Timmerman, L. Valentin, A. C. Testa, W. Froyman, C. Landolfo, A. Kotlarz, D. Fischerova, B. Van Calster, T. Bourne, Collaborators, L. Zanchi, M. Longo, F. Filippi, E. Farsi, J. Mafra, M. Beja, E. Facchetti, E. Panizzolo, V. Poonyakanok

**Affiliations:** ^1^ Gynecology and Obstetrics University Hospitals KU Leuven Leuven Belgium; ^2^ Development and Regeneration KU Leuven Leuven Belgium; ^3^ Department of Obstetrics and Gynecology Skåne University Hospital Malmö Sweden; ^4^ Department of Clinical Sciences Lund University Malmö Sweden; ^5^ Woman, Child and Public Health, Fondazione Policlinico Universitario A Gemelli IRCCS Rome Italy; ^6^ Obstetrics and Gynecology Università Cattolica del Sacro Cuore Rome Italy; ^7^ Queen Charlotte's and Chelsea Hospital, West London Gynaecological Cancer Centre Imperial College Healthcare NHS Trust London UK; ^8^ Department of Metabolism, Digestion and Reproduction, Faculty of Medicine Imperial College London London UK; ^9^ Department of Gynecology and Oncology, Faculty of Medicine Jagiellonian University Medical College Krakow Poland; ^10^ Department of Gynaecology, Obstetrics and Neonatology, First Faculty of Medicine Charles University and General University Hospital in Prague Prague Czech Republic; ^11^ Department of Biomedical Data Science Leiden University Medical Centre Leiden The Netherlands

**Keywords:** consensus, Doppler ultrasonography, gynecology, ovarian cyst, ovarian neoplasm, ultrasonography

## INTRODUCTION

The International Ovarian Tumor Analysis (IOTA) standardized terms, definitions and measurements to describe the ultrasound features of adnexal masses^1^ have now been adopted in clinical practice all over the world. They form the foundation for the prospective international multicenter IOTA studies, in which different methods to classify adnexal masses as benign or malignant or to predict risk of malignancy and type of malignancy were developed and validated^2^, ^3^, ^4^, ^5^, ^6^, ^7^, ^8^, ^9^.

The IOTA benign descriptors, the Assessment of Different NEoplasias in the adneXa (ADNEX) model and the two‐step strategy are recommended by the IOTA Steering Committee for the characterization of ovarian masses using ultrasound evaluation. These approaches allow a lesion to be assigned an Ovarian‐Adnexal Reporting and Data System (O‐RADS) category, as defined by the American College of Radiology^10^, and can be used to guide appropriate management^11^. Normal ovaries or functional findings are categorized as O‐RADS 1 and lesions to which an IOTA benign descriptor applies are classified as O‐RADS 2. If no benign descriptor applies, the ADNEX model is used to calculate the risk of malignancy, allowing classification into O‐RADS Categories 2–5 according to the calculated risk level.

From questions raised by participants in national and international congresses and courses, it is evident that some of the IOTA terms and definitions would benefit from updating. Correct use of this terminology is a prerequisite for the IOTA methods to work. Therefore, we have updated the terms and definitions about which users have expressed uncertainty.

Herein, we summarize the terms, definitions and measurements required for using the IOTA benign descriptors^9^, ADNEX model^6^ and two‐step strategy^9^ (Table 1), specifying the IOTA terms and definitions that we have newly added, updated or clarified (Table 2). This Consensus Statement is accompanied by a series of illustrative figures demonstrating key sonographic features and measurement principles, complemented with a series of videoclips (Videoclips [Link], [Link], [Link], [Link], [Link], [Link], [Link], [Link], [Link], [Link], [Link], [Link], [Link], [Link], [Link], [Link], [Link], [Link], [Link], [Link], [Link], [Link], [Link]), whose legends provide more detailed clinical and histological context.

**Table 1:** Terms, definitions and measurements required for using the International Ovarian Tumor Analysis (IOTA) benign descriptors and the Assessment of Different NEoplasias in adneXa (ADNEX) model

IOTA benign descriptors (BD)			
BD1	BD2	BD3	BD4
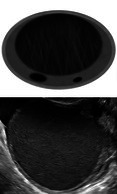	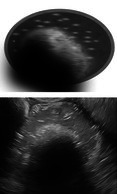	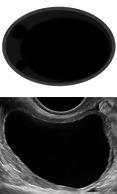	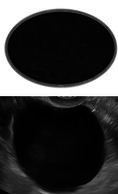
Unilocular cyst with ground‐glass echogenicity and largest diameter < 10 cm in a premenopausal woman (presumed diagnosis: endometrioma).	Unilocular cyst with mixed echogenicity and shadowing and largest diameter < 10 cm in a premenopausal woman (presumed diagnosis: dermoid cyst).	Unilocular cyst with regular walls, anechoic cyst fluid and largest diameter < 10 cm (presumed diagnosis: simple cyst or cystadenoma).	All other unilocular cysts with regular walls and largest diameter < 10 cm (classified as benign).

ADNEX	
Variable	Value
Maximum diameter of lesion	Value in mm
Maximum diameter of largest solid component	Value in mm
More than 10 cyst locules?	Yes/no
Number of papillary projections	0, 1, 2, 3 or ≥ 4
Acoustic shadows present?	Yes/no
Ascites present?	Yes/no

O‐RADS		
Category	Malignancy risk	Interpretation and management
O‐RADS 1	Normal ovary or functional changes (e.g. corpus luteal cyst)	Normal ovary, conservative management
O‐RADS 2	IOTA benign descriptors present or ADNEX risk of malignancy < 1%	Almost certainly benign lesion, conservative management is possible if no symptoms
O‐RADS 3	ADNEX risk of malignancy 1% to < 10%	Low risk of malignancy, management by local gynecologist
O‐RADS 4	ADNEX risk of malignancy 10% to < 50%	Intermediate risk of malignancy, management by gynecological oncologist
O‐RADS 5	ADNEX risk of malignancy 50% to 100%	High risk of malignancy, management by gynecological oncologist

O‐RADS, Ovarian‐Adnexal Reporting and Data System.

**Table 2:** International Ovarian Tumor Analysis (IOTA) terms and definitions to describe the ultrasound features of adnexal lesions that have been newly introduced, updated or clarified herein

Term	Definition
Solid component 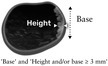	A solid component must have at least one diameter that is 3 mm or larger. Solid tissue with no diameter ≥ 3 mm is not classified as a solid component (Figure 4).
Papillary projection 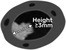	A papillary projection is a solid component protruding into the cyst cavity. It must have a height of ≥ 3 mm (Figure 4). If a protrusion of solid tissue with a height < 3 mm has any diameter of 3 mm or more (i.e. the maximum diameter of the base is 3 mm or more), then this protrusion is a solid component, but it is not a papillary projection.
Unilocular‐solid cyst 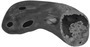	If a cyst with one cyst locule contains a solid component and the solid component contains microcysts, the mass is unilocular‐solid, not multilocular‐solid. Figure 8 shows a papillary projection with microcysts.
Papillary projections in solid masses 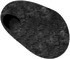	A solid tumor may have papillary projections protruding into a cystic area in the tumor. These papillary projections should be reported and counted. This is important when using an IOTA model that includes the variable ‘number of papillary projections’, such as the ADNEX model (0, 1, 2, 3, or ≥ 4) (Figure 9).
Irregular outer cyst wall 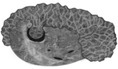	If the outer cyst wall is irregular because of solid tissue growing on the outside of the cyst, this is described as an irregular outer cyst wall (Figure 6m–o).
Irregular solid tumor 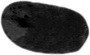	The presence of sharp angles (< 90°), lobulations or spiculated margins in the outer contour of a solid tumor classifies it as irregular. If a solid tumor contains any cysts with an irregularity in the inner cyst wall (e.g. papillary projections or other irregularities) or the contour of a cyst within a solid tumor is somewhat irregular, then the solid tumor is also described as irregular, even if its outer contour is smooth (Figure 10).
Variable cyst contents 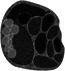	If different cyst locules in a multilocular cyst exhibit different echogenicity of cyst fluid (e.g. one locule contains anechoic fluid and two locules contain fluid with low‐level echogenicity), the cyst has variable cyst contents (Figure 12).
Acoustic shadows 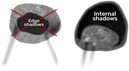	Edge shadowing is an artifact that may occur in any mass with a rounded outer contour. It is not reported as acoustic shadowing, because it does not arise from areas within the lesion (Figure 13). If attenuation causes a gradual decrease in signal intensity without a clearly defined area of signal loss, this should not be classified as an acoustic shadow, whereas internal shadowing reflects true loss of acoustic energy within the lesion.
Number of papillary projections 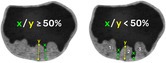	To distinguish a single papillary projection with multiple peaks from multiple separate papillary projections, for each adjacent pair of papillary peaks and the valley between them, the height of each peak and the height of the bottom of the valley are measured from the same baseline, perpendicular to the inner cyst wall or septum. If the height of the bottom of the valley is ≥ 50% of the height of the lower of the two adjacent peaks (measured from the same baseline), the two peaks are counted as a single papillary projection; if it is < 50% of the height of the lower peak, the two peaks are counted as two separate papillary projections. This assessment is repeated for each pair of adjacent peaks (Figure 17).
Number of cyst locules in a solid tumor 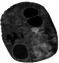	Once a mass is classified as a solid tumor (i.e. a tumor consisting of at least 80% solid tissue), the number of locules is not counted (Figure 19). When using the ADNEX model, the answer to the question, ‘More than 10 cyst locules?’ is always ‘No’ if the tumor is solid.
Color score 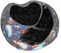	When assigning the color score, the highest degree of vascularization observed within the tumor should be considered (‘worst‐case scenario’ rule). For example, a color score of 4 is given if a tumor contains one highly vascularized solid component, even if the wall or septa or other solid components of the tumor show little vascularity (Figure 21m–o).
Type of center 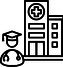	An oncology center is a tertiary referral center with a specific gynecological oncology unit. Examinations performed outside the oncology unit but within the same tertiary referral center, i.e. in patients not referred for gynecological oncology care, are classified as taking place in a non‐oncology center.

ADNEX, Assessment of Different NEoplasias in the adneXa.

## TERMS, DEFINITIONS AND MEASUREMENTS

The purpose of the IOTA terms is to ensure that there is a consistent approach to describing adnexal pathology using ultrasound, in both clinical and research contexts. While many of these terms and definitions are not required when using the IOTA benign descriptors or ADNEX model, they remain essential for consistent reporting, regardless of whether the operator applies a specific IOTA model.

### Lesion

An adnexal lesion is an adnexal mass inconsistent with normal physiology. It may represent part of an ovary, be separate from the ovary or completely replace the ovary so that any remaining normal ovarian tissue is no longer distinguishable. An adnexal mass consistent with normal physiology, for example, a hemorrhagic corpus luteal cyst, is not a lesion. If there is a persistent mass (i.e. still present after at least 3 months), for example, a unilocular cyst, surrounded by normal ovarian stroma with or without follicles, the whole ovary containing the cyst is the ‘ovary’, while the mass, for example, the unilocular cyst, is the ‘lesion’.

### Morphological features

#### Septum

**Figure 1:**
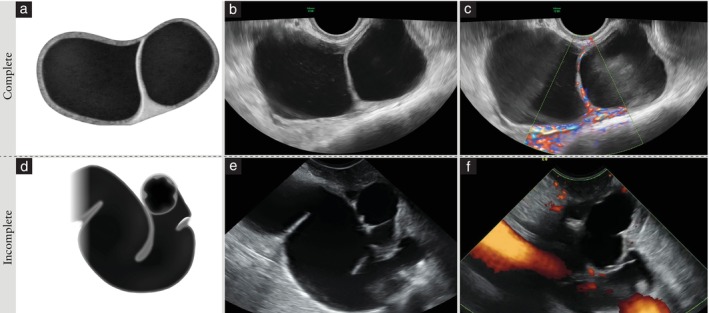
Complete and incomplete septa. Schematic drawings and grayscale and power Doppler ultrasound images showing: (a–c) a complete septum in a case of multilocular cyst, suggestive of mucinous cystadenoma; and (d–f) incomplete septa in a case of chronic hydrosalpinx. The incomplete hyperechogenic septa originate as triangular protrusions from the inner cyst wall, but do not reach the opposite wall. See also Videoclip S1 for more details.

A septum is defined as a thin strand of tissue running between two opposing inner walls of a cyst, or between the inner cyst wall and another septum, within a single lesion (Figure 1). Septa are found in multilocular or multilocular‐solid lesions.

#### Incomplete septum

An incomplete septum, as seen in hydrosalpinges, is defined as a thin strand of tissue that does not reach the opposite inner cyst wall in some scanning planes. If a cystic lesion has only incomplete septa and no solid component, it is classified as a unilocular cyst, even if, in some scanning planes, the cyst may look like a multilocular cyst (Figure 1).

#### Solid component

**Figure 2:**
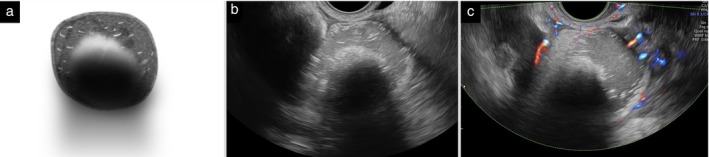
White ball. Schematic drawing (a) and grayscale (b) and power Doppler (c) ultrasound images showing a characteristic ‘white ball’ in a dermoid cyst. The white ball consists of hair and sebum and is not classified as a solid component. It is avascular on Doppler imaging (c). See also Videoclip S2 for more details.

**Figure 3:**
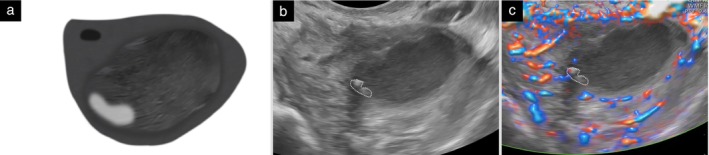
Sludge on the inner cyst wall. Schematic drawing (a) and grayscale (b) and power Doppler (c) ultrasound images showing sludge (encircled with dotted line) on the inner wall of an ovarian endometrioma. Sludge is not vascularized on power Doppler imaging (c). See also Videoclip S3 for more details.

A solid component is identified by echogenicity suggestive of solid tissue (such as myometrium or an ovarian fibroma). Blood clots and other amorphous material, for example, mucus or sebum, are not solid tissue and are not classified as solid components. This means that the hyper‐reflective and avascular area in a dermoid cyst (‘white ball’, consisting of hair and sebum) is not a solid component (Figure 2). ‘Sludge’ on the inner walls of endometriotic cysts is not a solid component (Figure 3). Cyst walls, septa (even thick septa) and normal ovarian stroma are also not classified as solid components of a tumor.

*How to discriminate between solid tissue and amorphous material*. Provided that Doppler settings are correctly optimized (see below under ‘Vascular features’), the detection of color Doppler signals within a structure confirms that the structure consists of solid tissue and so should be classified as a solid component (except septa, incomplete septa and normal ovarian stroma, which are not classified as solid components of a tumor even if vascularized on color Doppler). Absence of color Doppler signals does not exclude solid tissue, because this may reflect a technical issue. If in doubt, the structure should be classified as a solid component. A blood clot or a lump of mucus (that may be confused with solid tissue) never contains internal vascularity. In contrast to solid tissue, a blood clot often has a concave contour. A blood clot can be distinguished from solid tissue by pushing gently on the lesion with the ultrasound transducer or free hand. A blood clot usually exhibits a jelly‐like consistency, showing deformable movement under gentle probe pressure, whereas solid tissue remains firm. Blood clots or lumps of mucus may move away from the cyst wall when the lesion is pushed by the probe.

**Figure 4:**
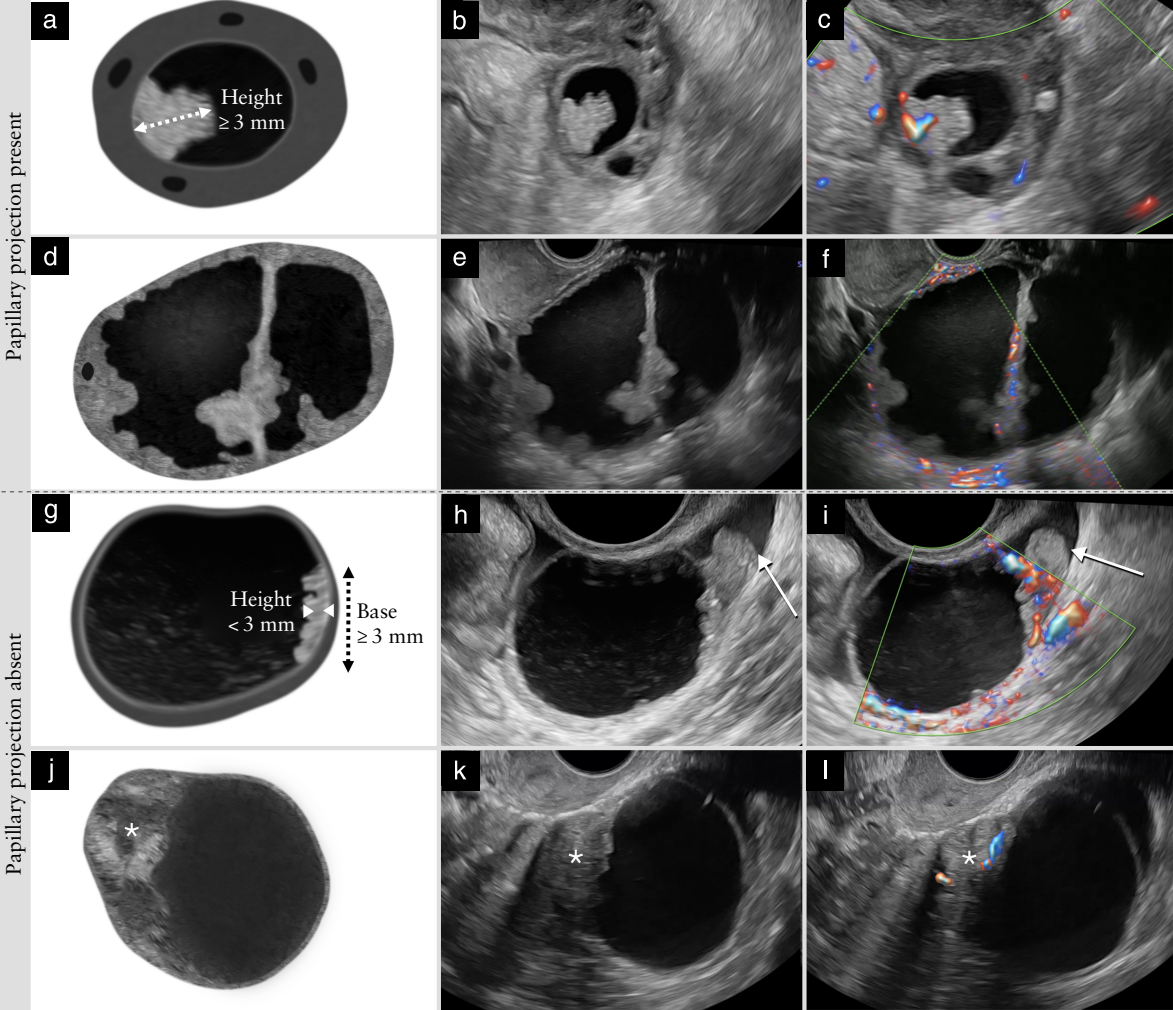
Papillary projection and other solid components. Schematic drawings and grayscale and power Doppler ultrasound images showing examples of solid components: (a–c) papillary projection, defined as protrusion of solid tissue into a cyst cavity with a height ≥ 3 mm; (d–f) papillary projections arising from both the septum and the cyst wall; (g–i) protrusion of solid tissue with a height < 3 mm and with at least one base ≥ 3 mm, i.e. a solid component that is not a papillary projection; and (j–l) solid tissue not protruding into the cyst cavity (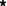), i.e. a solid component that is not a papillary projection. Arrow in (h,i) indicates the Fallopian tube. See also Videoclip S4 for more details.

**Updated definition**: A solid component must have at least one diameter that is 3 mm or larger. Solid tissue with no diameter ≥ 3 mm is not classified as a solid component (Figure 4).

#### Papillary projection

A solid component may protrude into the cyst cavity. A papillary projection is defined as solid tissue protruding into a cyst cavity with a height of at least 3 mm. There is no upper limit of size^1^, ^12^, ^13^.

A papillary projection can arise from the cyst wall or from a septum (in which case, its height is measured from the surface of the septum). A papillary projection may be the only solid component of a mass or the largest solid component of a mass. In either case, it should be recorded and measured both as a papillary projection and as the largest solid component of the mass. By definition, a papillary projection is a solid component, because it consists of solid tissue. However, a solid component that either does not protrude into the cyst cavity or protrudes into the cyst cavity with a height < 3 mm is not a papillary projection (Figure 4).

**Clarification**: A papillary projection is a solid component protruding into the cyst cavity. It must have a height of ≥ 3 mm (Figure 4). If a protrusion of solid tissue with a height < 3 mm has any diameter of 3 mm or more (i.e. the maximum diameter of the base is 3 mm or more), then this protrusion is a solid component, but it is not a papillary projection.

**Figure 5:**
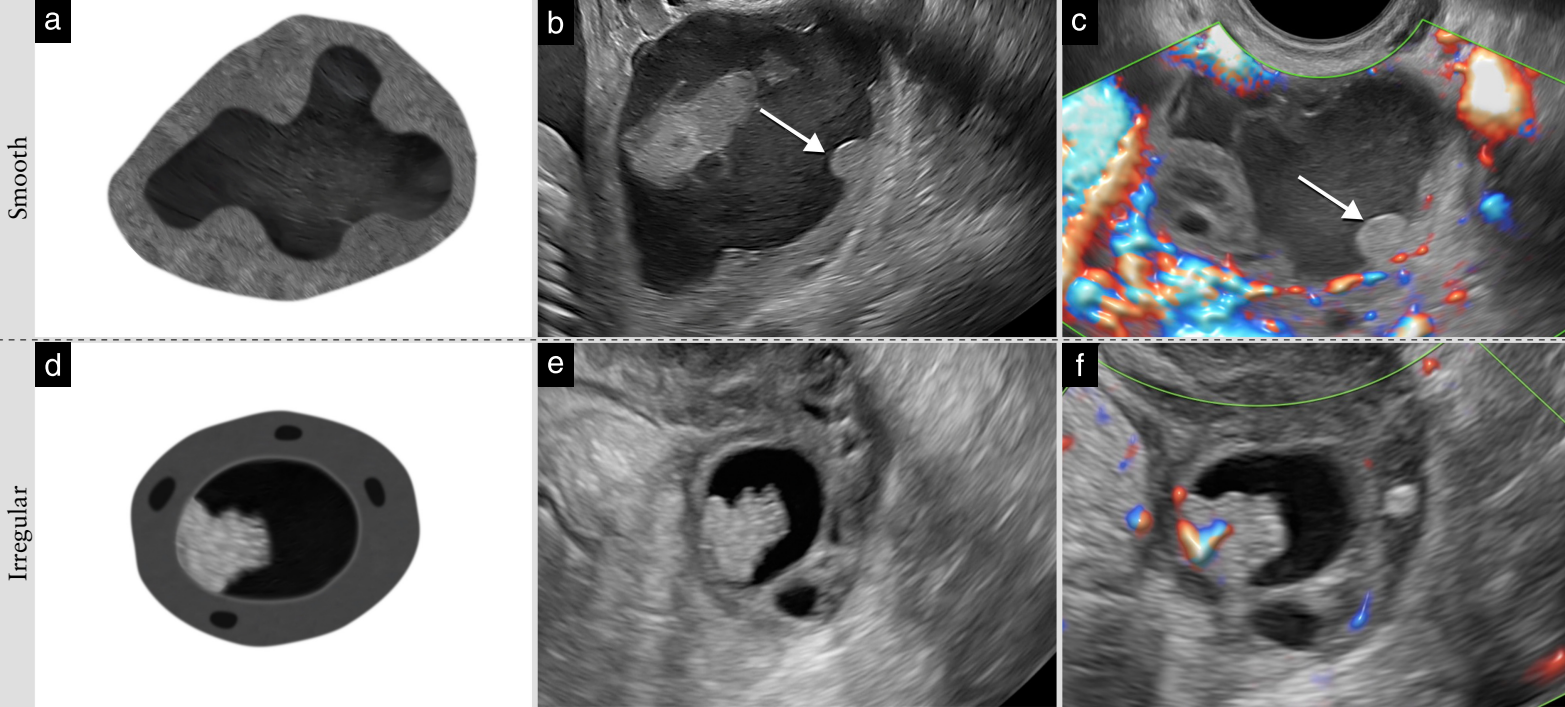
Papillary projection surface. Schematic drawings and grayscale and power Doppler ultrasound images showing papillary projections with: (a–c) smooth surface; and (d–f) irregular surface. In (b,c), the smooth surface of the papillary projection is indicated with an arrow. See also Videoclip S5 for more details.

*Papillary projection surface (contour)*. The surface (i.e. contour) of a papillary projection is described as ‘smooth’ or ‘irregular’ (i.e. cauliflower‐like) (Figure 5).

#### Inner and outer cyst walls

**Figure 6:**
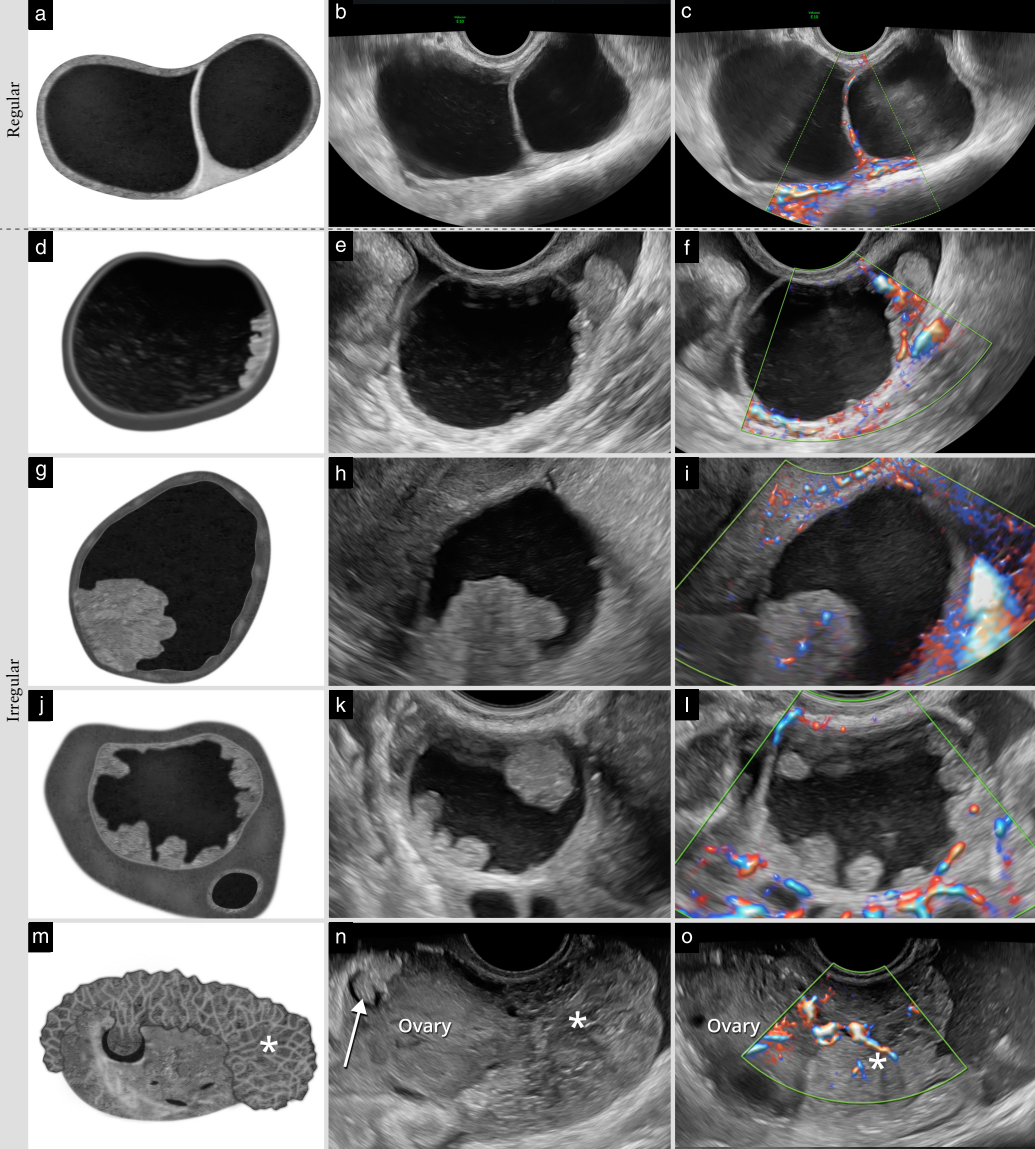
Regular or irregular cyst wall. Schematic drawings and grayscale and power Doppler ultrasound images showing: (a–c) regular inner cyst walls; and (d–o) irregular inner cyst walls. In (d–f), protrusions of solid tissue with a height < 3 mm can be seen (wall irregularities, not papillary projections). In (g–i), the wall is irregular due to a single papillary projection. In (j–l), the wall is irregular due to multiple papillary projections. In (m–o), the unilocular‐solid cyst has an irregular inner wall due to a single papillary projection (arrow) and an irregular outer wall due to solid tumor tissue arising from the cyst and protruding externally through the ovarian capsule (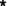). See also Videoclip S6 for more details.

The inner cyst wall is the inner lining of the cyst, which may be regular (smooth) or irregular (Figure 6). The inner cyst wall is considered irregular whenever solid tissue protrudes into the cyst cavity. If the protrusion has a height < 3 mm, it is classified as a wall irregularity; if it has a height ≥ 3 mm, it is classified as a papillary projection. By definition, the presence of a papillary projection always renders the inner cyst wall irregular, even if the surface of the projection itself is smooth. If the walls appear irregular because of sludge or hair, the inner cyst walls may be perceived as irregular, but if it is apparent that the ‘irregularity’ is explained by sludge or hair, then the wall should be classified as regular.

When fluid is present in the pelvis, the outer cyst wall may also be assessed and described as irregular when solid tissue is seen growing on its external surface (Figure 6m–o).

**Updated definition**: If the outer cyst wall is irregular because of solid tissue growing on the outside of the cyst, this is described as an irregular outer cyst wall (Figure 6m–o).

#### Type of lesion

**Figure 7:**
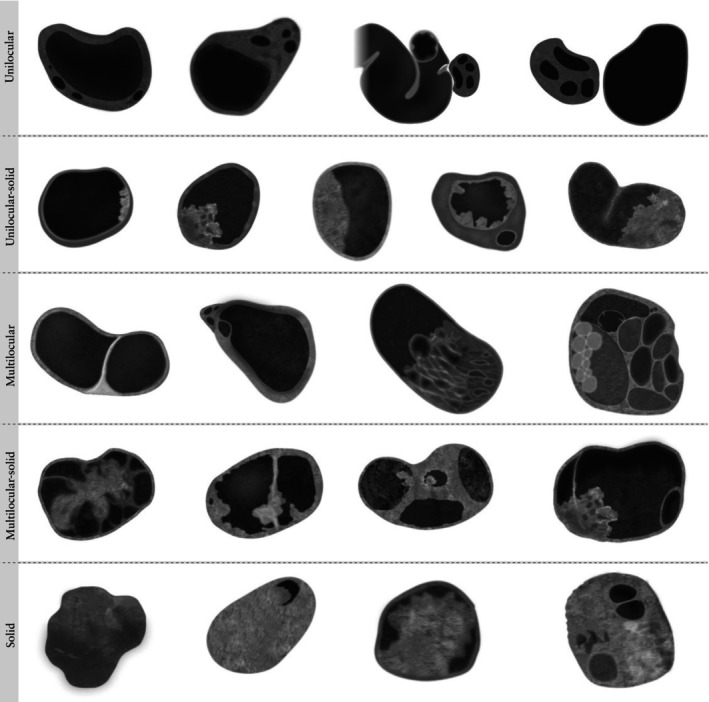
Types of lesion. Schematic drawings giving examples of different types of lesion: unilocular cyst, unilocular‐solid cyst, multilocular cyst, multilocular‐solid cyst and solid tumor. See also Videoclip S7 for more details.

Lesions are classified into six categories (Figure 7): unilocular cyst, unilocular‐solid cyst, multilocular cyst, multilocular‐solid cyst, solid tumor and ‘not classifiable’.

*Unilocular cyst*. A unilocular cyst has one cyst locule, i.e. is a cyst without septa and without any solid components (no papillary projections or other solid components). Normal ovarian stroma is not regarded as a ‘solid part’ of a tumor. For example, a peritoneal cyst with one cyst locule containing a normal ovary is classified as a unilocular cyst and not as a unilocular‐solid cyst (provided that the ultrasound examiner is certain that the solid tissue is an ovary).

*Unilocular‐solid cyst*. A unilocular‐solid cyst has one cyst locule and one or more solid components. The solid component may be a papillary projection or another type of solid component. A solid component has at least one diameter of 3 mm or more. A pyosalpinx or hydrosalpinx with classic features of the cogwheel sign (Figure S1) or beads‐on‐a‐string sign^14^ (Figure S2) might fall into this category if any diameter of the protrusions into the cyst cavity is ≥ 3 mm.

**Figure 8:**
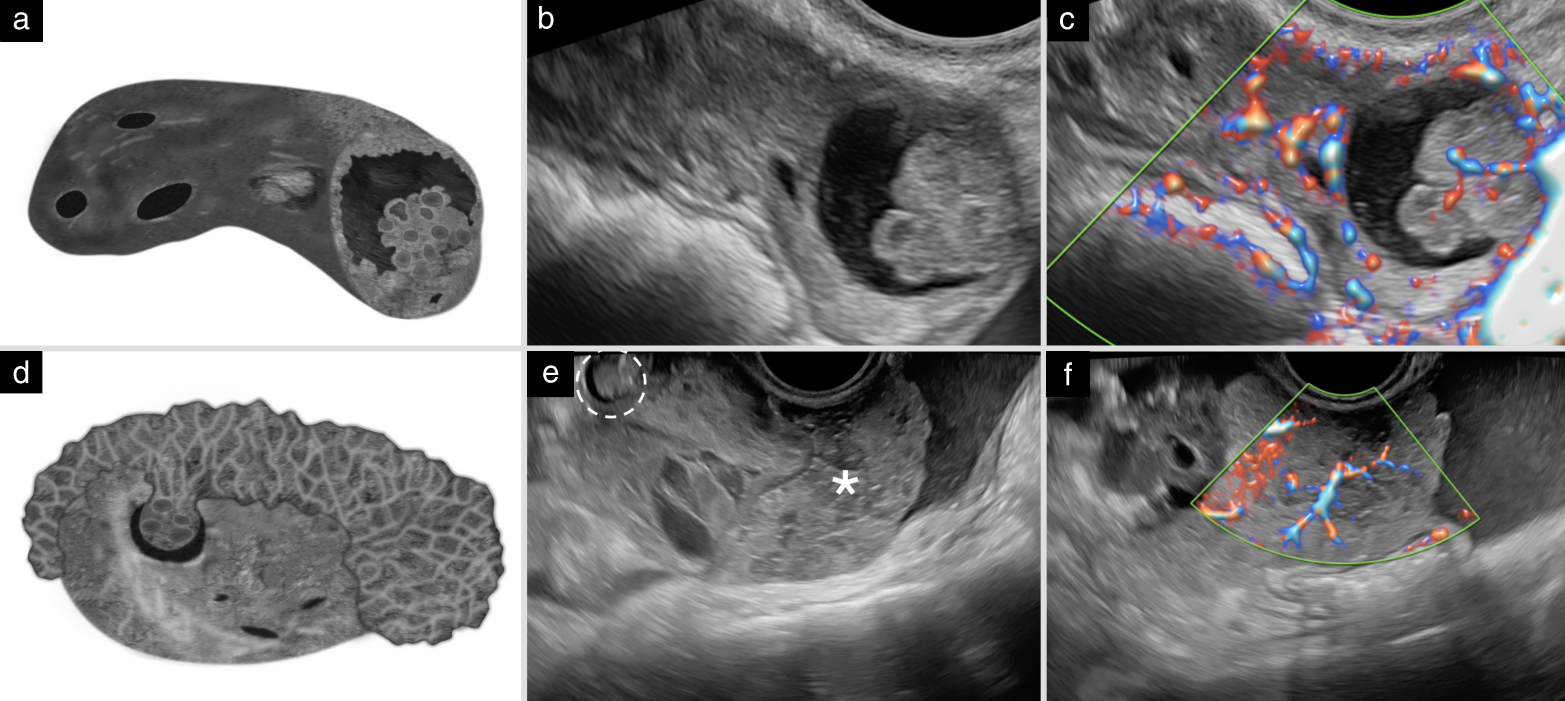
Microcystic pattern. Schematic drawings and grayscale and power Doppler ultrasound images showing a solid component with microcysts (microcystic pattern). (a–c) Unilocular‐solid cyst with a papillary projection containing microcysts. (d–f) Ovary containing a unilocular‐solid cyst with a single papillary projection (dashed circle), in which the solid tumor tissue forming the papillary projection demonstrates both intracystic growth into the cyst cavity and exophytic growth extending through the ovarian capsule, showing a microcystic pattern (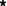). See also Videoclip S8 for more details.

**Updated definition:** If a cyst with one cyst locule contains a solid component and the solid component contains microcysts, the mass is unilocular‐solid, not multilocular‐solid. Figure 8 shows a papillary projection with microcysts.

*Multilocular cyst*. A multilocular cyst has at least one septum, but no solid components. A cyst with small ‘daughter cysts’ is also multilocular.

*Multilocular‐solid cyst*. A multilocular‐solid cyst has at least one septum and one or more solid components.

*Solid tumor*. A solid tumor is one in which the solid components comprise 80% or more of the total tumor volume on scanning (i.e. on assessment in two‐dimensional scanning planes throughout the whole tumor volume).

*Not classifiable*. A cyst is deemed not classifiable because of poor visualization, for example, because of strong acoustic shadowing due to calcification.

#### Papillary projections in solid masses

**Figure 9:**
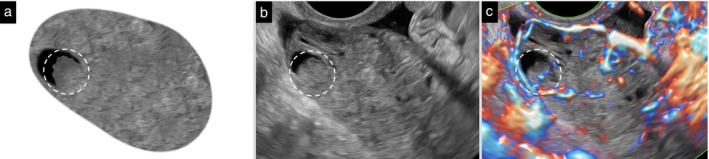
Papillary projection in solid tumor. Schematic drawing (a) and grayscale (b) and power Doppler (c) ultrasound images showing a solid tumor with a small cyst containing a papillary projection. Dashed circle surrounds the papillary projection. See also Videoclip S9 for more details.

**Updated definition:** A solid tumor may have papillary projections protruding into a cystic area in the tumor. These papillary projections should be reported and counted. This is important when using an IOTA model that includes the variable ‘number of papillary projections’, such as the ADNEX model (0, 1, 2, 3, or ≥ 4) (Figure 9).

#### Irregular solid tumor

**Figure 10:**
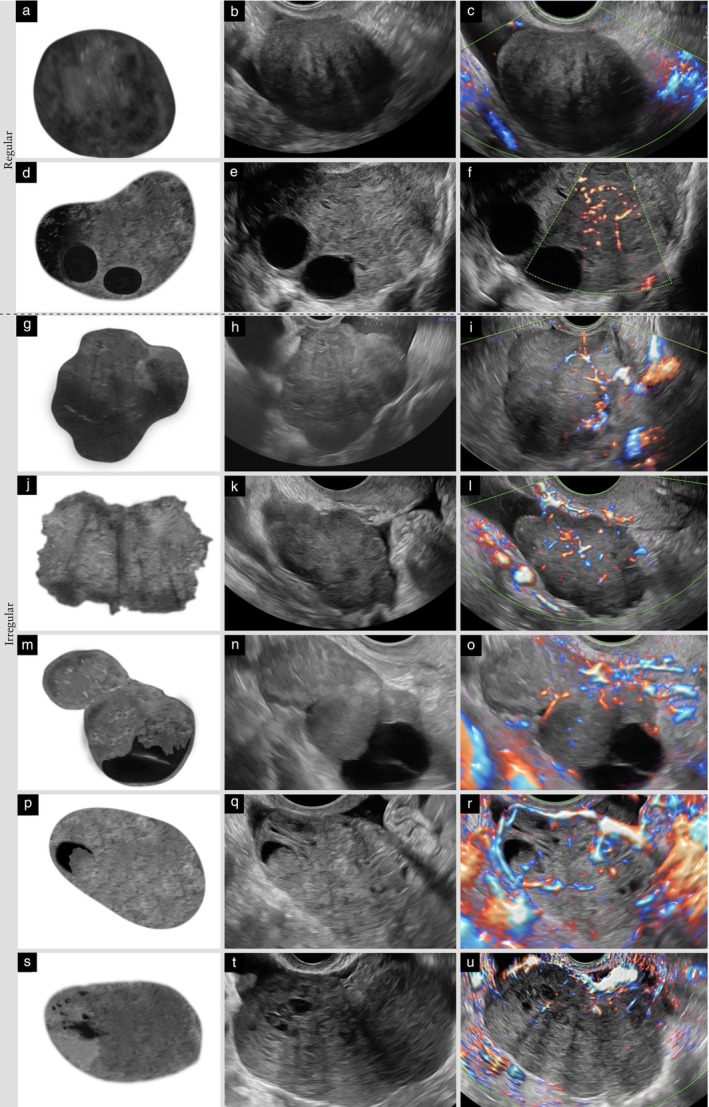
Regular or irregular solid tumor. Schematic drawings and grayscale and power Doppler ultrasound images showing examples of: (a–f) regular solid tumors; and (g–u) irregular solid tumors. (a–c) Round regular solid tumor. (d–f) Solid tumor with a regular outer contour and two internal cysts with regular inner cyst walls. (g–i) Solid tumor with an irregular gently lobulated surface. (j–l) Solid tumor with spiculated contour. (m–o) Irregular solid tumor showing one sharp angle of the outer wall. (p–r) Oval solid tumor with regular outer contour, but with a small internal cyst with a papillary projection inside (i.e. irregular solid tumor). (s–u) Solid tumor with regular outer contour, but multiple internal cysts with irregular contour (i.e. irregular solid tumor). See also Videoclip S10 for more details.

**Updated definition**: The presence of sharp angles (< 90°), lobulations or spiculated margins in the outer contour of a solid tumor classifies it as irregular. If a solid tumor contains any cysts with an irregularity in the inner cyst wall (e.g. papillary projections or other irregularities) or the contour of a cyst within a solid tumor is somewhat irregular, then the solid tumor is also described as irregular, even if its outer contour is smooth (Figure 10).

#### Echogenicity of cyst contents

**Figure 11:**
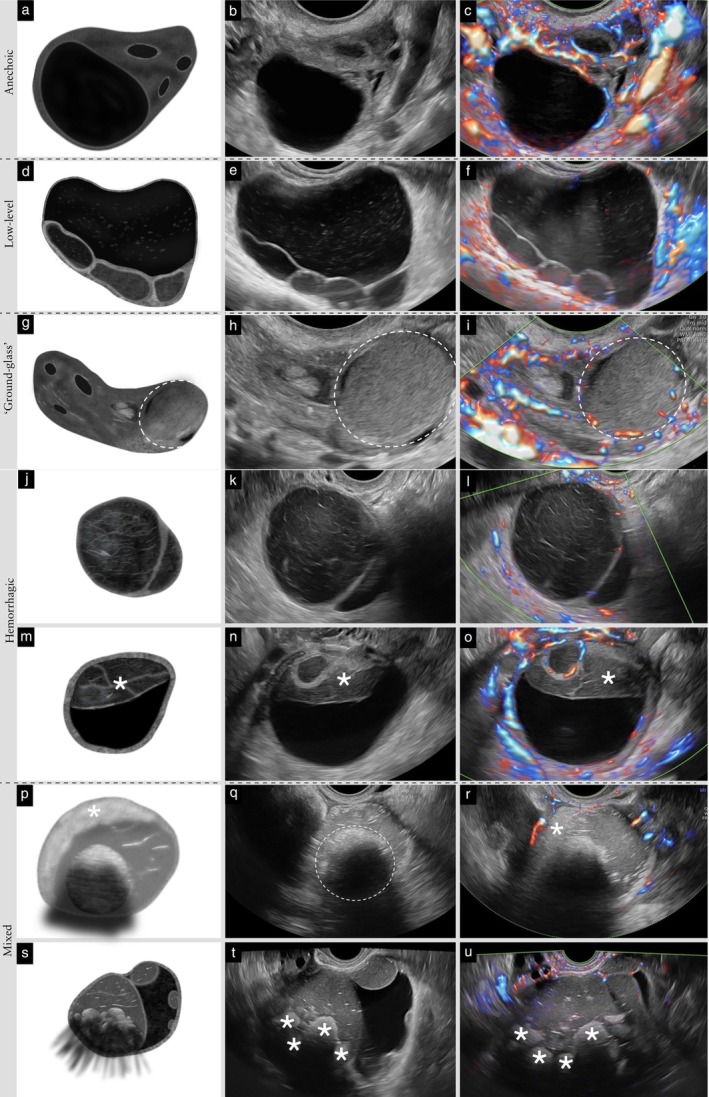
Echogenicity of cyst contents. Schematic drawings and grayscale and power Doppler ultrasound images illustrating different types of cyst contents: (a–c) anechoic; (d–f) low‐level echogenicity; (g–i) ‘ground‐glass’ echogenicity; (j–o) hemorrhagic; and (p–u) mixed echogenicity. In (g–i), the dashed line encircles an intraovarian lesion with ground‐glass echogenicity. In (j–l), the hemorrhagic cyst contains fibrin strands (‘cobweb‐like’ appearance). In (m–o), the hemorrhagic cyst contains a blood clot (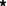). In (p–u), the mixed echogenicity is suggestive of a dermoid cyst, with thin echogenic linear (often horizontal) structures and bright echogenic spots (both corresponding to hair), a ‘white ball’ (dashed circle), the ‘mushroom‐cap’ sign (single 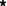 in (r)) and ‘cotton‐wool tufts’ (multiple 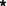 in (t,u)). See also Videoclip S11 for more details.

The echogenicity of cyst contents of a lesion is described as: anechoic (completely black); low‐level echogenicity (a few scattered echoes); ‘ground‐glass’ echogenicity (the typical homogeneous echogenicity seen in endometriotic cysts)^14^; hemorrhagic (thread‐like structures representing fibrin strands; blood clots; star‐shaped or cobweb‐like echogenicity; a blood clot may move in a jelly‐like fashion if pushed upon with the probe); or mixed echogenicity (contents with different types of echogenicity in the same cyst locule, as seen in a dermoid cyst) (Figure 11)^15^.

**Figure 12:**
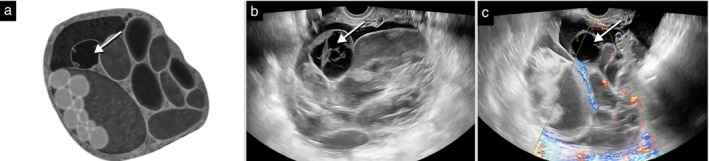
Variable cyst contents. Schematic drawing (a) and grayscale (b) and power Doppler (c) ultrasound images showing a multilocular cyst with different echogenicity of cyst fluid in different locules. Some cyst locules contain anechoic contents (arrows) and others contain fluid with low‐level echogenicity. See also Videoclip S12 for more details.

**New definition**: If different cyst locules in a multilocular cyst exhibit different echogenicity of cyst fluid (e.g. one locule contains anechoic fluid and two locules contain fluid with low‐level echogenicity), the cyst has variable cyst contents (Figure 12).

#### Acoustic shadows

An acoustic shadow is the result of loss of acoustic echoes posterior to a structure that absorbs or reflects the ultrasound beam. Acoustic shadows may be observed behind a round white ball in dermoid cysts and behind fibrous papillary projections or calcifications. Fan‐shaped shadowing is typically seen in ovarian fibromas (and uterine leiomyomas). Importantly, acoustic shadows arise from areas within the lesion, not from its edges.

**Figure 13:**
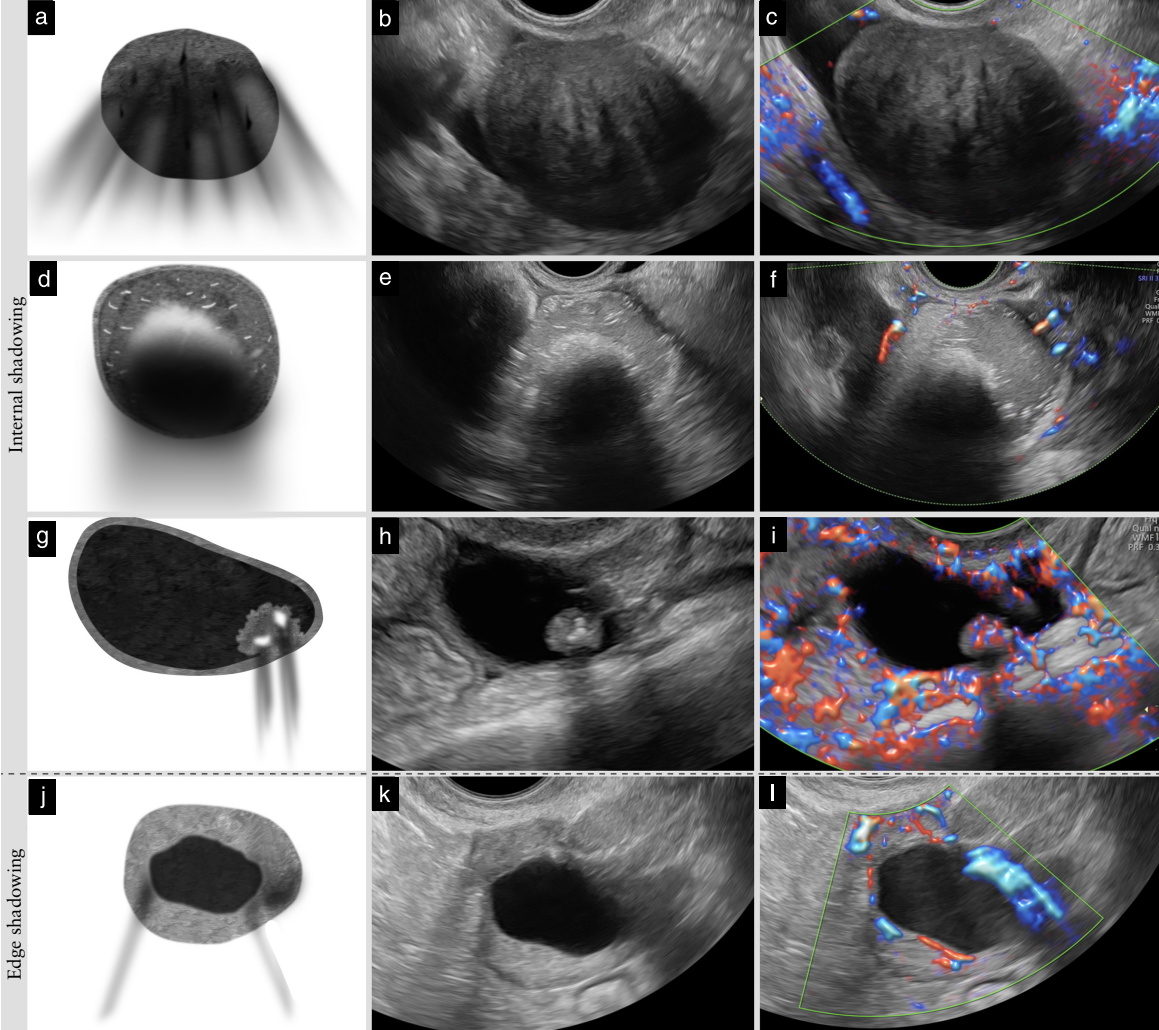
Types of shadowing. Schematic drawings and grayscale and power Doppler ultrasound images illustrating different types of shadowing: (a–c) fan‐shaped shadowing in a solid tumor; (d–f) shadowing behind a ‘white ball’ in a dermoid cyst; (g–i) shadowing behind calcifications in a papillary projection in a unilocular‐solid cyst; and (j–l) edge shadowing from a unilocular cyst (the latter is not classified as acoustic shadows). See also Videoclip S13 for more details.

**Updated definition**: Edge shadowing is an artifact that may occur in any mass with a rounded outer contour. It is not reported as acoustic shadowing, because it does not arise from areas within the lesion (Figure 13). If attenuation causes a gradual decrease in signal intensity without a clearly defined area of signal loss, this should not be classified as an acoustic shadow, whereas internal shadowing reflects true loss of acoustic energy within the lesion.

#### Ascites

**Figure 14:**
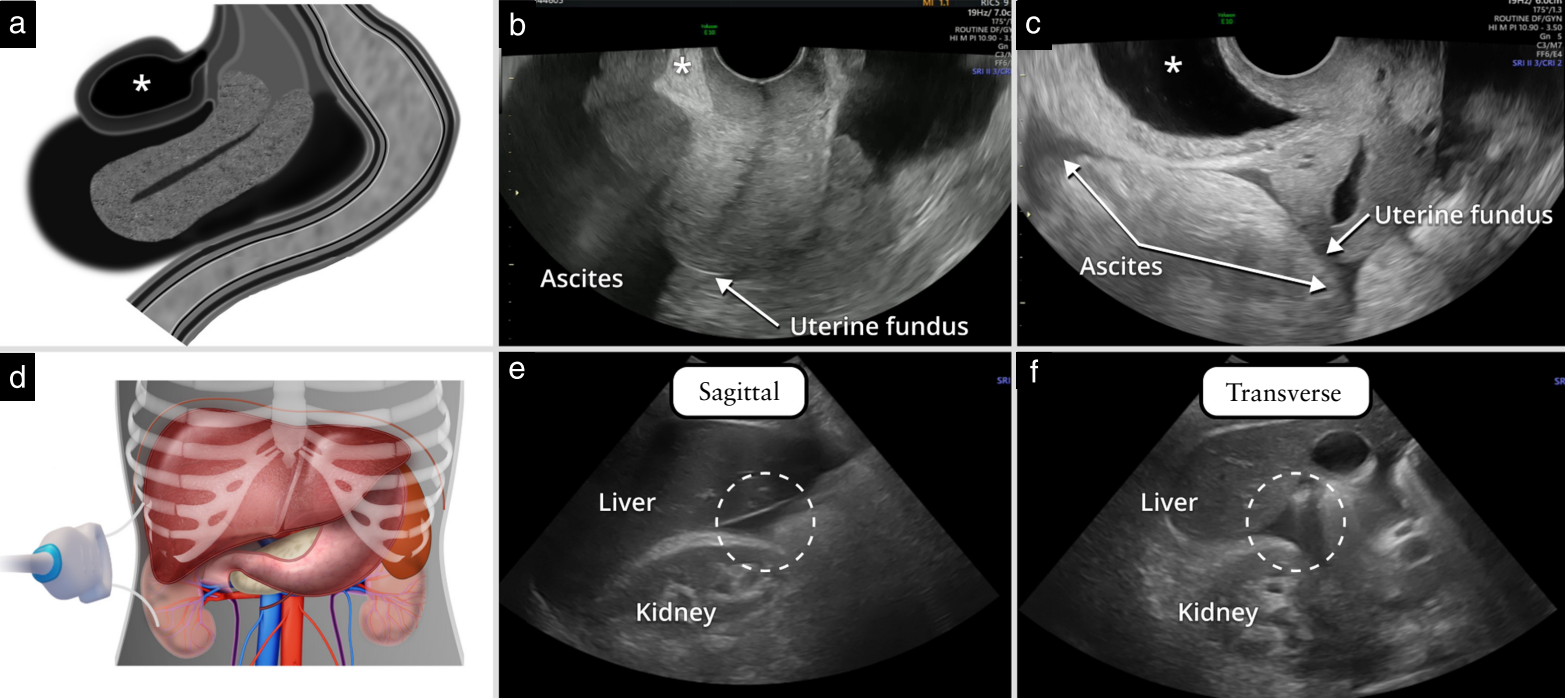
Ascites. (a–c) Schematic drawing (a) and transvaginal grayscale ultrasound images from two different patients (b,c), illustrating ascites with fluid above the level of the uterine fundus. The location of the urinary bladder is indicated (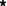). (d–f) Schematic drawing (d) showing the transabdominal approach for evaluation of Morrison's pouch, and transabdominal ultrasound images showing fluid in Morrison's pouch (encircled with dashed line) in sagittal (e) and transverse (f) planes (same patient as in (c)). See also Videoclip S14 for more details.

Ascites is defined as fluid outside the pouch of Douglas (PoD). Ascites is present if fluid is seen between the bladder and the uterus or above the level of the uterine fundus on transvaginal ultrasound, or in the Morrison's pouch (the intraperitoneal space between the right liver lobe and the right kidney)^16^ on transabdominal ultrasound (Figure 14).

### Type of center

The ADNEX model includes the predictor ‘type of center’, which can be either an oncology center or another type of center.

**New definition**: An oncology center is a tertiary referral center with a specific gynecological oncology unit. Examinations performed outside the oncology unit but within the same tertiary referral center, i.e. in patients not referred for gynecological oncology care, are classified as taking place in a non‐oncology center.

The main reason to include this variable as a predictor is to obtain more reliable risk estimates (i.e. better calibration performance). The prevalence of malignant tumors is higher among patients seen in oncology centers than that in patients seen in other centers. Without including type of center, the estimated risk of malignancy will be too low, on average, for patients in oncology centers and too high for patients in other centers. This is supported by the findings of Van Calster *et al*.^8^, who showed that, compared with the calibration of the ADNEX model, the calibration of logistic regression model 2 (LR2), which does not include type of center as a predictor, was poorer both in oncology centers and in other centers.

### Measurements and quantitative assessment of morphology

#### Lesion

**Figure 15:**
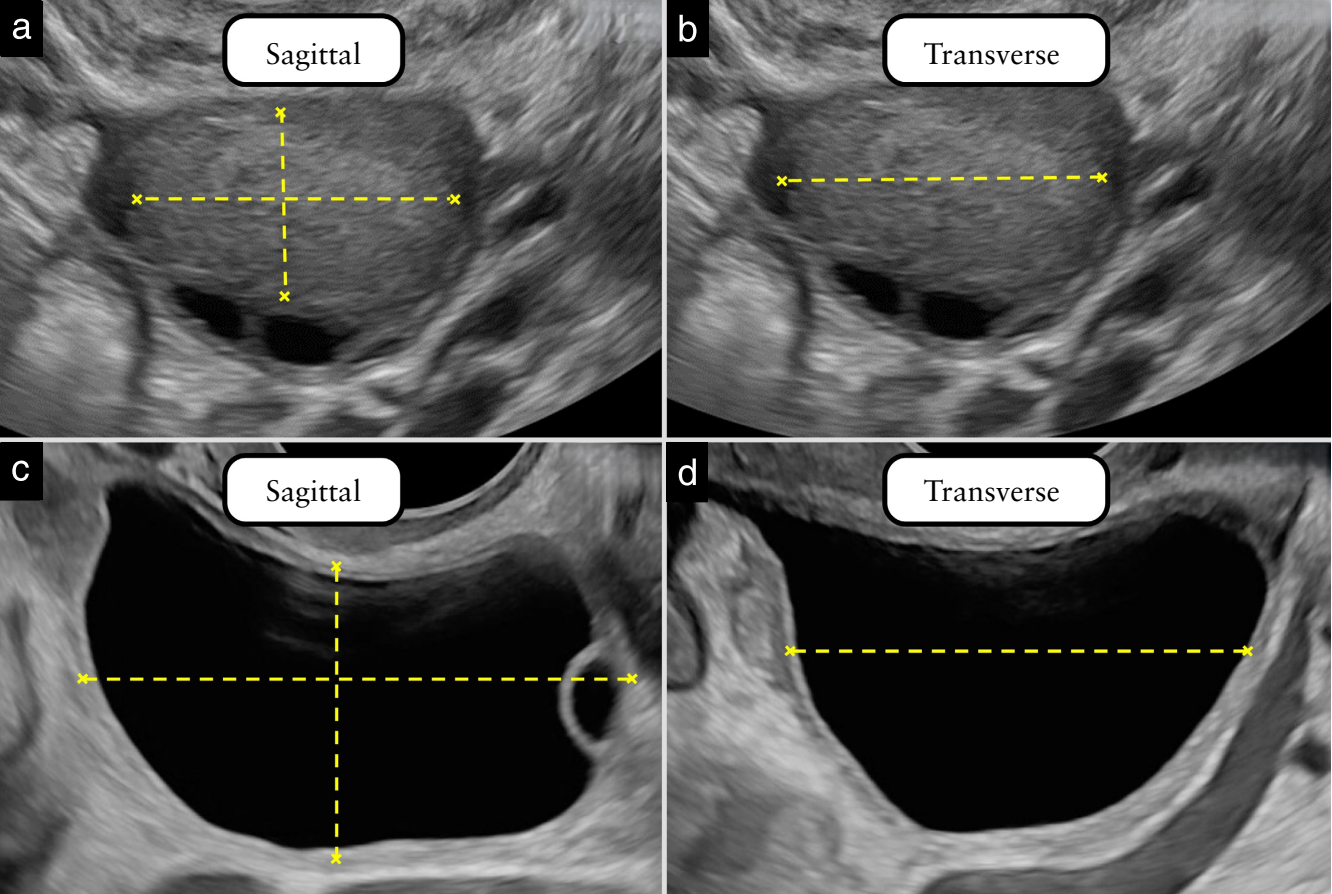
Lesion measurement: examples showing measurement of a unilocular cyst with ground‐glass content (a,b) and a multilocular cyst with anechoic content (c,d). The length and anterior–posterior diameters of a lesion are measured perpendicular to each other in a sagittal section through the cyst at the point at which the lesion appears to be at its largest (a,c). Then, the transducer is rotated 90°, to obtain a transverse section through the lesion, and its maximum transverse diameter is measured (b,d). If the cyst capsule is visible, as seen in (c,d), it is included in the measurement of the lesion. See also Videoclip S15 for more details.

The size of the ovaries, lesion(s) and solid components (including papillary projections) is measured (in mm) in two perpendicular planes, and three orthogonal diameters are measured (Figure 15). The measurements are taken where the structure to be measured (lesion/ovary/solid component) appears to be at its largest. If there is a lesion outside the ovary (e.g. hydrosalpinx), the ovary and the lesion are measured separately. If the lesion is located within the ovary, but can be distinguished clearly from the surrounding ovarian tissue, the lesion and the ovary are measured separately. If the lesion and the ovary are undistinguishable, they are measured as a single mass.

#### Papillary projection

**Figure 16:**
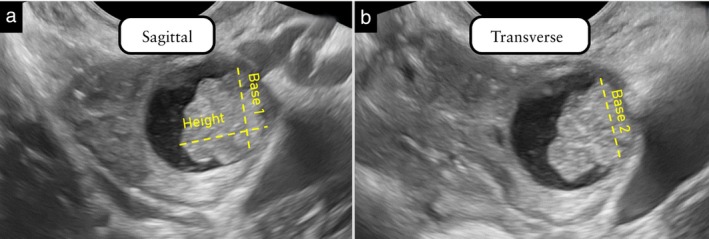
Measurement of a papillary projection. The height and Base 1 of a papillary projection are measured in the sagittal section through the lesion in which the papillary projection appears to be largest (a). The thickness of the cyst wall from which the papillary projection arises is not included in the height measurement. Then, the transducer is rotated 90°, to obtain a transverse section through the lesion. The maximum transverse diameter (Base 2) of the papillary projection is measured in this section (b). If there is more than one papillary projection, the largest is measured. See also Videoclip S16 for more details.

The largest papillary projection is measured in two perpendicular planes, and three orthogonal diameters are measured: height, Base 1 and Base 2 (Figure 16). The thickness of the wall or of the septum from which the papillary projection arises must not be included in the measurement of papillary height.

#### Solid component

In unilocular‐solid and multilocular‐solid cysts, the largest solid component is measured (three orthogonal diameters in two perpendicular planes). In some lesions, a papillary projection might be the largest solid component.

#### Number of papillary projections

If papillary projections are confluent, it can be difficult to count the number of papillary projections.

**Figure 17:**
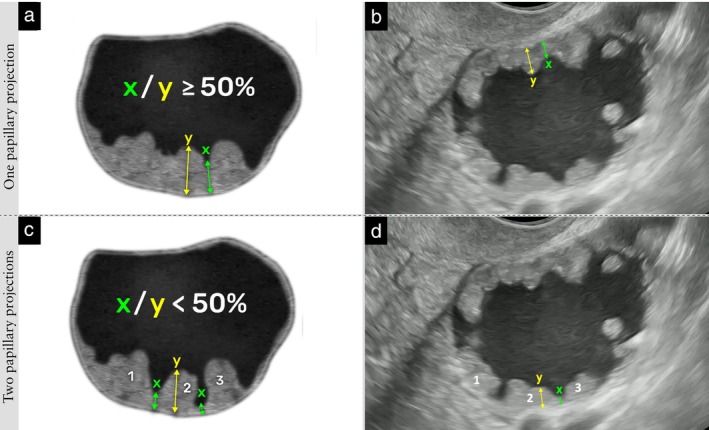
Assessment of number of papillary projections. Schematic drawings and ultrasound images showing how to distinguish a single papillary projection with multiple peaks from multiple separate papillary projections. The x/y ratio is assessed for each adjacent pair of papillary peaks and the valley between them. This assessment is performed repeatedly when multiple papillary projections are present. (a,b) For an adjacent pair of peaks, if ‘x’ (height of the bottom of the valley between two adjacent peaks) divided by ‘y’ (height of the lower of the two peaks, measured from the same baseline) is ≥50%, the adjacent pair of peaks is counted as a single papillary projection. (c,d) If x/y is <50%, the adjacent pair of peaks is counted as two separate papillary projections; thus, three papillary projections are present in this example (marked 1–3). See also Videoclip S17 for more details.

**Updated definition**: To distinguish a single papillary projection with multiple peaks from multiple separate papillary projections, for each adjacent pair of papillary peaks and the valley between them, the height of each peak and the height of the bottom of the valley are measured from the same baseline, perpendicular to the inner cyst wall or septum. If the height of the bottom of the valley is ≥ 50% of the height of the lower of the two adjacent peaks (measured from the same baseline), the two peaks are counted as a single papillary projection; if it is < 50% of the height of the lower peak, the two peaks are counted as two separate papillary projections. This assessment is repeated for each pair of adjacent peaks (Figure 17).

#### Number of cyst locules

**Figure 18:**
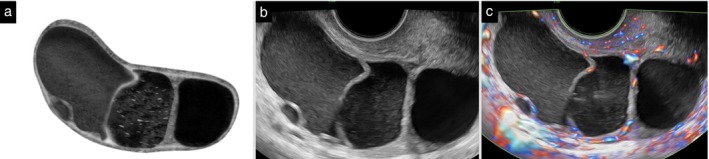
Assessment of number of cyst locules. Schematic drawing (a) and grayscale (b) and power Doppler (c) ultrasound images of a multilocular cyst containing four cyst locules. See also Videoclip S18 for more details.

The number of cyst locules is counted in the whole volume of the tumor, with the exception of solid tumors (Figure 18). In the ADNEX model, the exact number of locules is not needed; only whether there are more than 10 cyst locules (yes or no) is noted.

**Figure 19:**
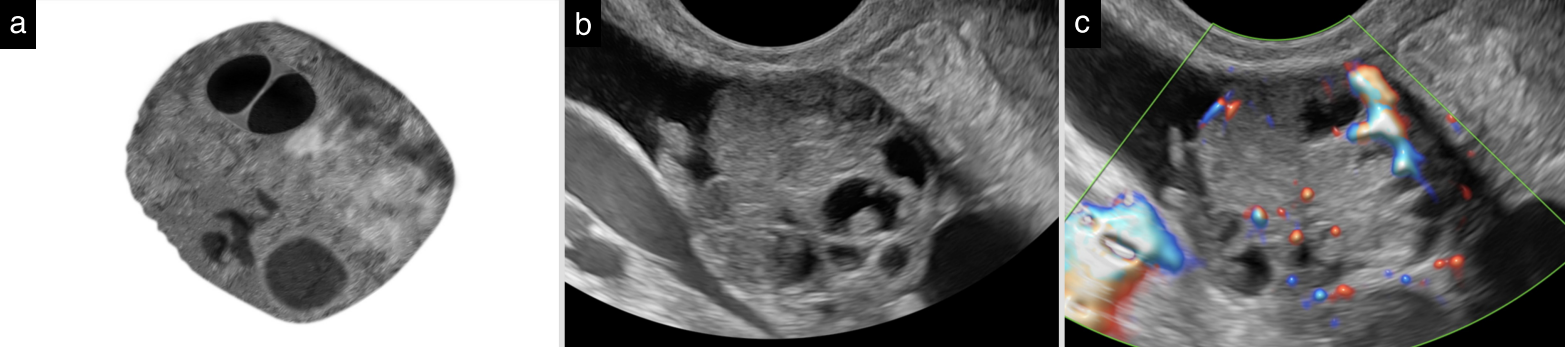
Solid tumor with internal cysts. Schematic drawing (a) and grayscale (b) and power Doppler (c) ultrasound images of a solid tumor with multiple internal cysts. These cysts are not counted because the tumor is solid, i.e. it consists of at least 80% solid tissue. See also Videoclip S19 for more details.

**Updated definition:** Once a mass is classified as a solid tumor (i.e. a tumor consisting of at least 80% solid tissue), the number of locules is not counted (Figure 19). When using the ADNEX model, the answer to the question, ‘More than 10 cyst locules?’ is always ‘No’ if the tumor is solid.

#### Fluid in the pouch of Douglas

In the original IOTA consensus on the terms, definitions and measurements of the sonographic features of adnexal tumors^1^, fluid in the PoD was quantified by measuring the largest anteroposterior diameter of the PoD in the sagittal plane. This approach is no longer included in this updated consensus opinion, as quantification of pelvic free fluid has limited clinical relevance; assessment is now based solely on the presence or absence of ascites.

### Vascular features

**Figure 20:**
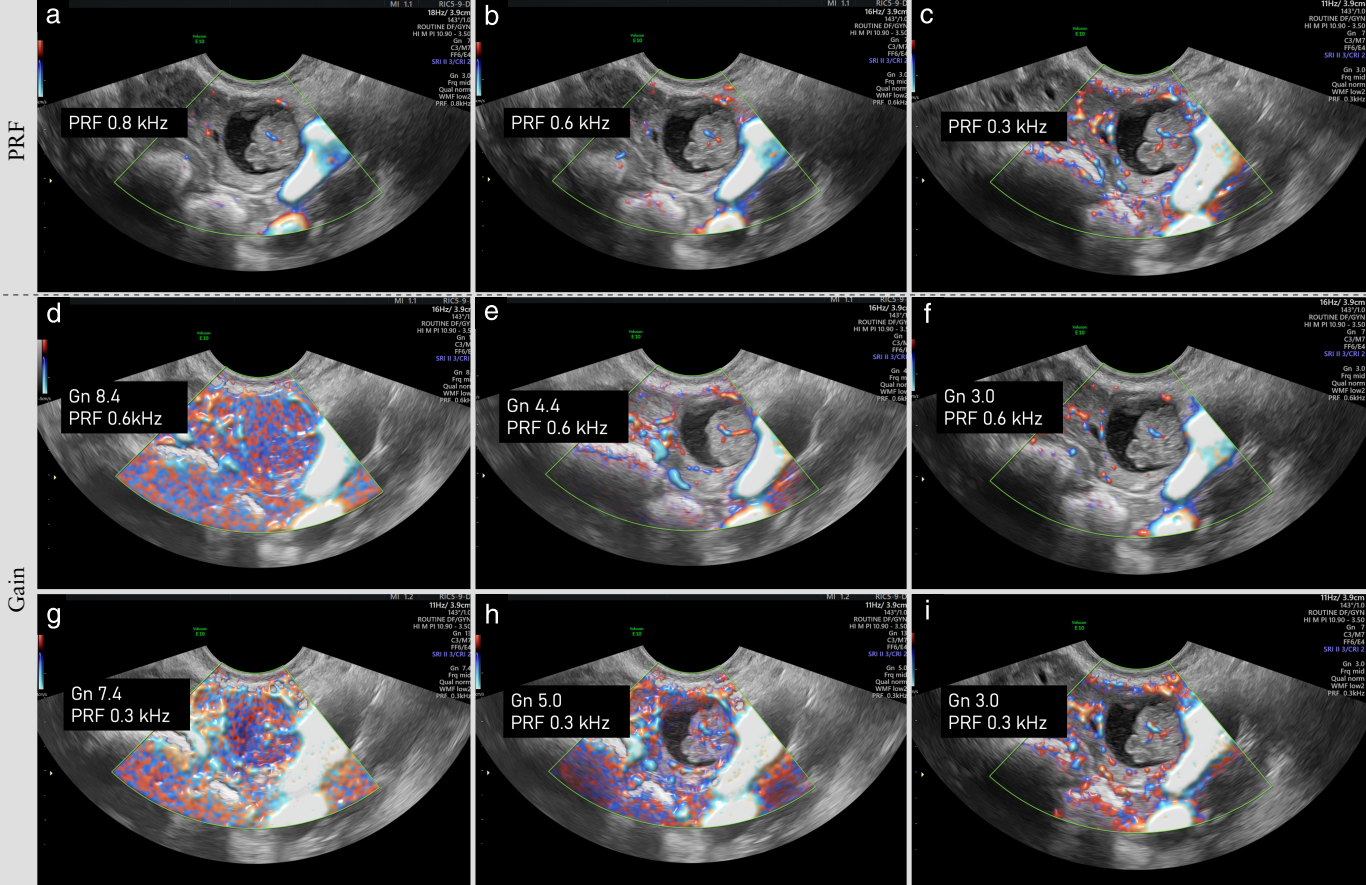
Doppler settings. Power Doppler ultrasound images of a unilocular‐solid cyst with a papillary projection obtained with different pulse repetition frequency (PRF) and power Doppler gain (Gn): (a–c) fixed gain (Gn, 3.0) and decreasing PRF, from 0.8 kHz (a) to PRF 0.6 kHz (b) to PRF 0.3 kHz (c); (d–f) fixed PRF (PRF, 0.6 KHz) and decreasing Gn, from 8.4 (d) to 4.4 (e) to 3.0 (f); (g–i) fixed PRF (PRF, 0.3KHz) and decreasing Gn, from 7.4 (g) to 5.0 (h) to 3.0 (i). See also Videoclip S20 for more details.

The entire tumor, including wall, septa and solid components, should be examined with color Doppler or power Doppler, using a pulse repetition frequency of 0.3–0.6 kHz (corresponding to a velocity scale of 3–6 cm/s). The color or power Doppler gain should be maximized without generating Doppler artifacts. We recommend starting with a high color gain and then gradually decreasing this until any Doppler artifacts disappear (Figure 20).

When using transabdominal ultrasound, the Doppler settings should be adjusted according to the body mass index of the patient and the distance from the probe to the tumor, to maximize detection of Doppler signals without artifacts.

Either color Doppler or power Doppler may be used, depending on the Doppler sensitivity of the ultrasound equipment and the preference of the examiner. New Doppler technologies allow visualization of very small blood vessels with low blood‐flow velocities (microvascularization). The IOTA color score should not be assessed using these new techniques.

#### Color score

**Figure 21:**
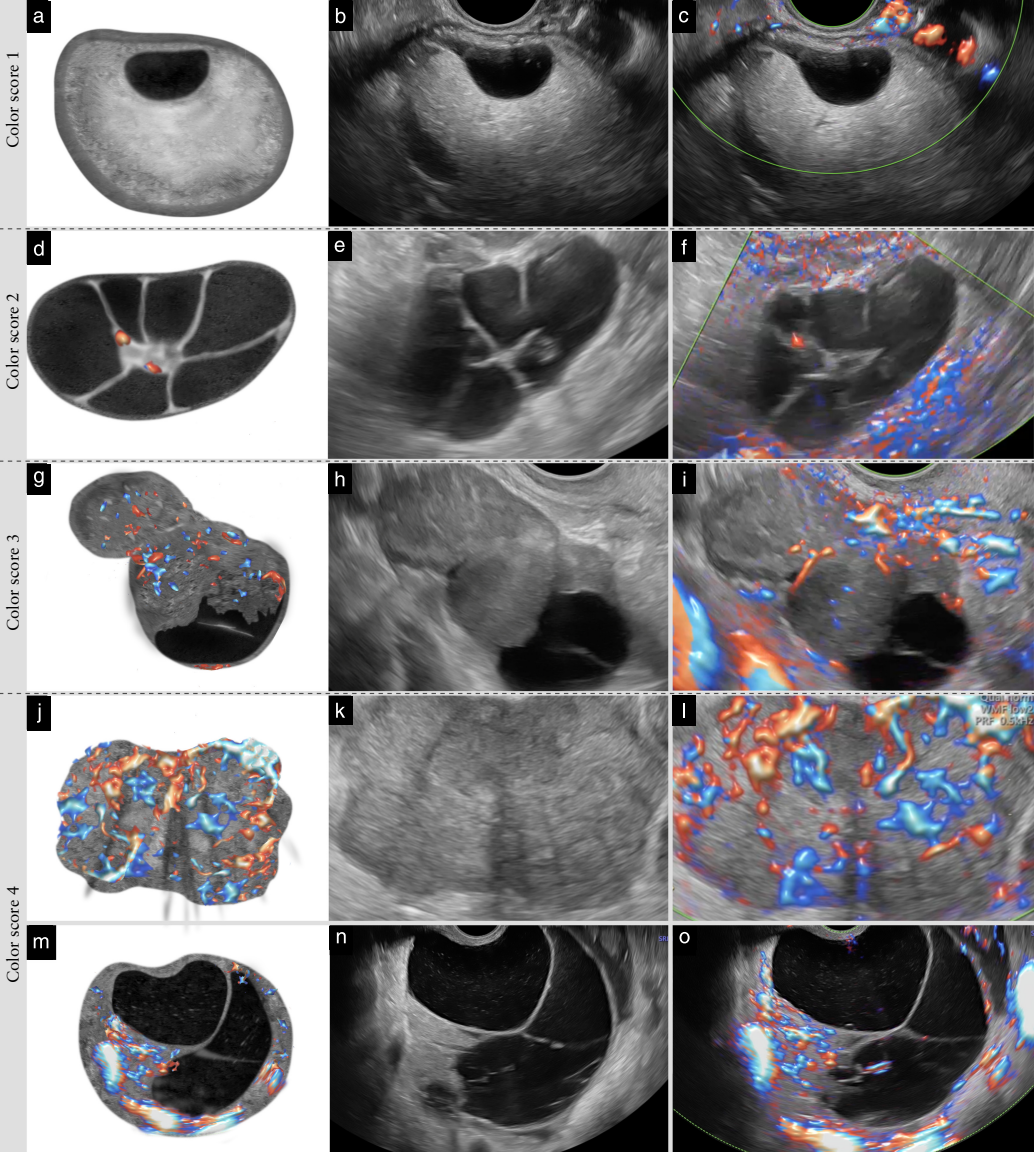
Assessment of the color content of a tumor. Schematic drawings and grayscale and power Doppler ultrasound images showing: (a–c) tumor with color score 1; (d–f) tumor with color score 2; (g–i) tumor with color score 3; and (j–l) tumor with color score 4. (m–o) A color score of 4 is given for a tumor with one highly vascularized solid component, even if the wall and septa of the tumor are only minimally or moderately vascularized or no color is detectable in them (‘worst‐case scenario’ rule). See also Videoclip S21 for more details.

Blood flow in papillary projections is classified as present or absent. If a subjective semiquantitative assessment of tumor vascularization is made, the following terms should be used to describe the vascularization of the septa, cyst walls and solid components: a color score of 1 is given when no color or power Doppler signals can be detected in the lesion; a color score of 2 is given when only a minimal amount of color or power Doppler signals can be detected; a color score of 3 is given when a moderate amount of color or power Doppler signals is present; and a color score of 4 is given when abundant color or power Doppler signals are present (Figure 21). The color score refers to the color content of the scan, not the Doppler shift spectrum. It is given for the tumor as a whole.

**Updated definition**: When assigning the color score, the highest degree of vascularization observed within the tumor should be considered (‘worst‐case scenario’ rule). For example, a color score of 4 is given if a tumor contains one highly vascularized solid component, even if the wall or septa or other solid components of the tumor show little vascularity (Figure 21m–o).

### IOTA terms: a quick guide

**Figure 22:**
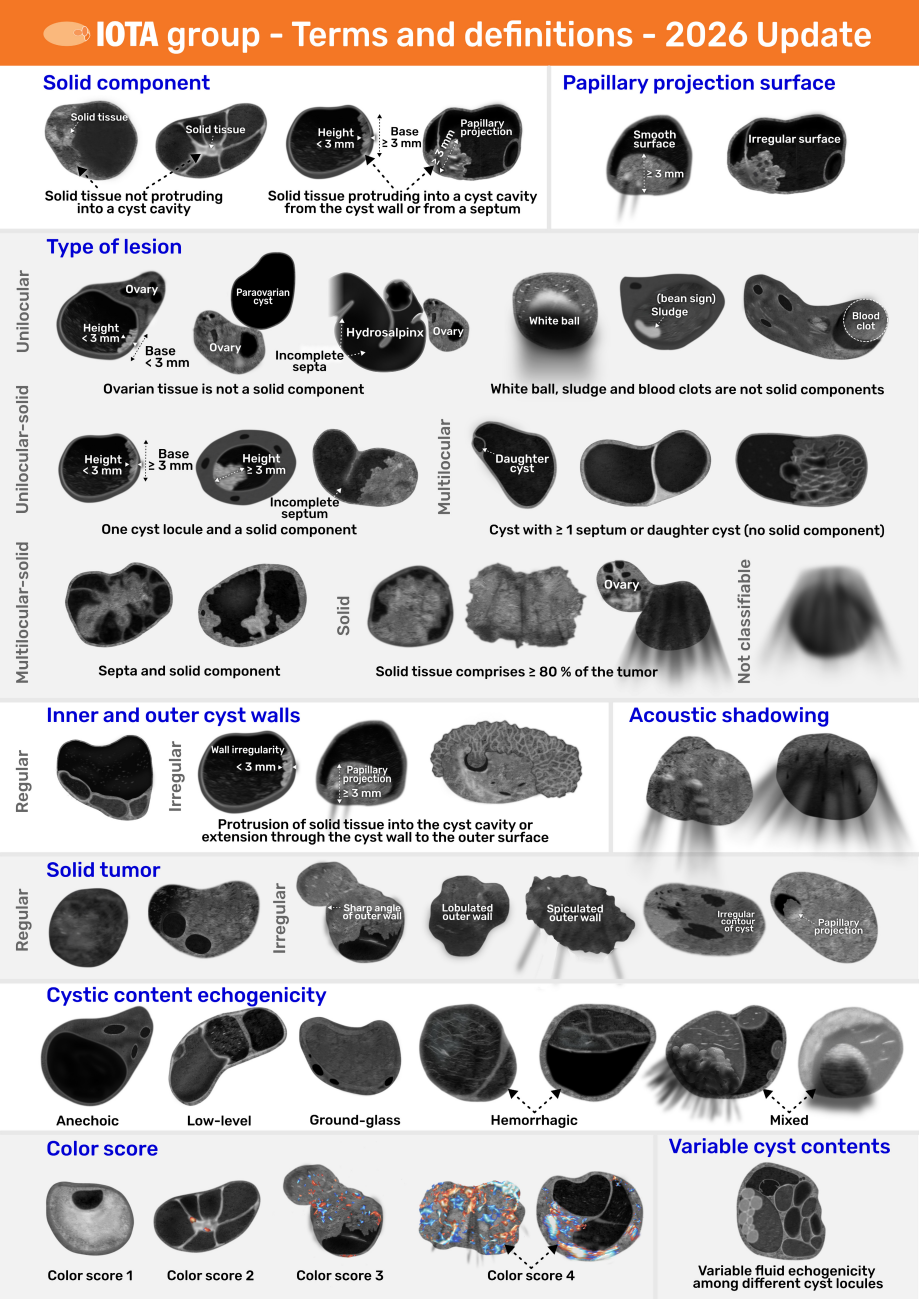
Overview of updated International Ovarian Tumor Analysis (IOTA) terms and definitions to describe the ultrasound features of adnexal lesions.

Figure 22 provides a visual overview of these standardized terms and definitions, as an easy‐to‐use reference for daily clinical practice.

## Supporting information


**Figure S1** Cogwheel sign. (a) Schematic drawing showing the cogwheel sign (encircled by dashed line), which is caused by swollen mucosal folds protruding into the lumen of a fluid‐filled, inflamed Fallopian tube. Grayscale (b) and power Doppler (c) ultrasound images of an inflamed Fallopian tube filled with fluid of low‐level echogenicity. In longitudinal section, incomplete septa (pseudosepta) are seen, corresponding to folding (‘kinking’/doubling‐up) of the distended tube, resulting in a unilocular cystic appearance. In cross‐section, thickened mucosal folds (_*_) produce the characteristic cogwheel appearance (encircled by dashed lines). See also Videoclip S22.

**Figure S2** Beads‐on‐a‐string sign. (a) Schematic drawing showing the beads‐on‐a‐string sign (encircled by dashed line) in the cross‐section of a Fallopian tube. Grayscale (b) and power Doppler (c) ultrasound images of a chronic hydrosalpinx, showing tiny protrusions into the fluid‐filled tube, with ‘beads‐on‐a‐string’ appearance in cross‐section (encircled by dashed line). The tiny protrusions correspond to fibrotic, flattened endosalpingeal folds. See also Videoclip S23.

**Videoclip S1** Grayscale and power Doppler ultrasound imaging showing a complete septum and incomplete septa. Case 1 shows a complete septum in a patient with a multilocular cyst (suggestive of mucinous cystadenoma). Case 2 shows incomplete septa, defined as a thin band of tissue that originates as a triangular protrusion from one of the walls, but does not reach the opposite inner cyst surface (chronic hydrosalpinx exhibiting the beads‐on‐a‐string sign).

**Videoclip S2** Grayscale and power Doppler ultrasound imaging showing a characteristic ‘white ball’ in a dermoid cyst. The white ball consists of hair and sebum and is not classified as a solid component. It is avascular on Doppler imaging.

**Videoclip S3** Grayscale and power Doppler ultrasound imaging showing sludge on the inner wall of an ovarian endometrioma. Sludge is not vascularized on power Doppler imaging.

**Videoclip S4** Grayscale and power Doppler ultrasound imaging showing examples of papillary projections and non‐papillary solid components. Cases 1 and 2 demonstrate papillary projections, defined as protrusions of solid tissue into a cyst cavity with a height ≥ 3 mm. In Case 1, the patient was diagnosed with a histologically confirmed serous borderline ovarian tumor, FIGO Stage IA; in Case 2, showing a papillary projection on the septum, the diagnosis was a histologically confirmed serous borderline ovarian tumor, FIGO Stage IIIA2. Case 3 (confirmed cystadenofibroma in a paraovarian cyst) shows a protrusion of solid tissue with a height < 3 mm and a base ≥ 3 mm, which qualifies as a solid component because at least one diameter of the protrusion is ≥ 3 mm, but is not a papillary projection. Case 4 (clear cell ovarian carcinoma arising in an endometrioma, FIGO Stage IA) shows a solid component that does not protrude into the cyst cavity and, therefore, does not meet the definition of a papillary projection.

**Videoclip S5** Grayscale and power Doppler ultrasound imaging showing papillary projections with smooth or irregular surface. Case 1 shows a unilocular‐solid cyst with ‘ground‐glass’ echogenicity and a smooth papillary projection in a 15‐week‐pregnant patient, with a diagnosis of decidualized ovarian endometrioma confirmed at follow‐up imaging. Case 2 shows a cyst with an irregular papillary projection (histologically confirmed serous borderline ovarian tumor, FIGO Stage IA).

**Videoclip S6** Grayscale and power Doppler ultrasound imaging showing regular and irregular inner and outer cyst walls. Case 1 shows a multilocular cyst with regular inner cyst wall (suggestive of mucinous cystadenoma). Case 2 shows a unilocular‐solid cyst with an irregular inner cyst wall, due to the presence of a protrusion of solid tissue into the cyst cavity (wall irregularity with height < 3 mm, but base ≥ 3 mm) (histologically confirmed paraovarian serous cystadenofibroma). Case 3 shows a unilocular‐solid cyst with an irregular inner cyst wall, due to a single papillary projection (histologically confirmed serous borderline ovarian tumor, FIGO Stage IA). Case 4 shows a unilocular‐solid tumor with an irregular inner cyst wall, due to the presence of multiple papillary projections (histologically confirmed bilateral serous borderline ovarian tumor, FIGO Stage IB). Case 5 displays a unilocular‐solid cyst with an irregular inner wall, caused by the presence of a single papillary projection, and an irregular outer wall, due to the presence of solid tumor tissue arising from the cyst, which has a disrupted capsule (histologically confirmed serous borderline ovarian tumor, FIGO Stage IC2).

**Videoclip S7** Grayscale and power Doppler ultrasound imaging showing different types of lesion: unilocular cyst, unilocular‐solid cyst, multilocular cyst, multilocular‐solid cyst, solid tumor and a non‐classifiable lesion. Case 1 shows a unilocular cyst (suggestive of functional cyst). Case 2 shows a unilocular‐solid cyst with a papillary projection with acoustic shadowing (edge and internal shadowing) and moderate perfusion of the papillary projection on power Doppler imaging (histologically confirmed serous borderline ovarian tumor, FIGO Stage IA). Case 3 shows a unilocular‐solid cyst with an incomplete septum and richly vascularized solid tissue (histologically confirmed high‐grade serous carcinoma of the Fallopian tube, FIGO Stage IIIC). Case 4 shows a multilocular cyst with low‐level echogenicity of cyst contents and moderately vascularized septa (histologically confirmed mucinous borderline ovarian tumor, FIGO Stage IA). Case 5 shows an intraovarian multilocular cyst with a daughter cyst and low‐level echogenicity of cyst contents (suggestive of functional cyst or cystadenoma). Case 6 shows a multilocular‐solid cyst with a large central solid component entrapped within locules and abundant perfusion on power Doppler imaging (histologically confirmed ovarian metastasis from endometrioid endometrial carcinoma, FIGO Stage IIIA). Case 7 shows a round solid tumor arising from the left ovary, with fan‐shaped shadowing (image suggestive of ovarian fibroma). Case 8 shows a lesion that is not classifiable due to poor visualization because of strong shadowing (image suggestive of ovarian fibroma; using a lower frequency would probably have improved the image and made the lesion classifiable).

**Videoclip S8** Grayscale and power Doppler ultrasound imaging showing a solid component with microcysts (microcystic pattern). Case 1 shows a unilocular‐solid cyst with a papillary projection containing microcysts (histologically confirmed serous borderline ovarian tumor, FIGO Stage IA). Case 2 shows an ovary containing a unilocular‐solid cyst with a single papillary projection demonstrating both intracystic and exophytic growth of the solid tissue with a microcystic pattern (histologically confirmed serous borderline ovarian tumor, FIGO Stage IC2).

**Videoclip S9** Grayscale and power Doppler ultrasound imaging showing a solid tumor with a small cyst containing a papillary projection (histologically confirmed high‐grade serous carcinoma of the Fallopian tube, FIGO Stage IVB).

**Videoclip S10** Grayscale and power Doppler ultrasound imaging showing examples of regular and irregular solid tumors. Case 1 shows a round regular solid tumor with a smooth surface and fan‐shaped shadowing (histologically confirmed ovarian fibroma). Case 2 shows an oval regular solid tumor with a smooth surface and two regular internal cysts (histologically confirmed ovarian metastasis from gastric cancer). Cases 3–7 show irregular solid tumors. Case 3 shows an irregular solid tumor with a gently lobulated surface (histologically confirmed high‐grade serous carcinoma, FIGO Stage IIA). Case 4 shows an irregular solid tumor with a spiculated surface (histologically confirmed ovarian metastases from primary breast cancer). Case 5 shows an irregular solid tumor with two sharp angles of the outer wall (histologically confirmed ovarian fibroma with focal ovarian stromal hyperplasia). Case 6 shows an irregular solid tumor with smooth surface, but a small internal cyst containing a papillary projection (histologically confirmed high‐grade serous carcinoma of the Fallopian tube, FIGO Stage IVB). Case 7 shows an oval irregular solid tumor with smooth surface, but multiple internal cysts with irregular contour (histologically confirmed ovarian fibroma with ascites, Meigs' syndrome).

**Videoclip S11** Grayscale and power Doppler ultrasound imaging showing different echogenicities of cyst contents. Case 1 shows anechoic cyst fluid in a unilocular cyst (functional cyst on follow‐up). Case 2 shows a multilocular cyst with low‐level echogenicity of cyst fluid (suggestive of mucinous cystadenoma). On power Doppler imaging, acoustic streaming is visible as movement of echogenic particles away from the transducer in the direction of wave propagation. Case 3 shows a unilocular cyst with ‘ground‐glass’ content (ovarian endometrioma on follow‐up). Cases 4 and 5 show cysts with hemorrhagic cyst contents: fibrin strands, i.e. cobweb‐like appearance, in Case 4 and blood clot in Case 5 (functional hemorrhagic cysts on follow‐up). Cases 6 and 7 show cysts with mixed echogenicity of cyst fluid (suggestive of dermoid cysts): thin echogenic linear (often horizontal) structures and bright echogenic spots (both corresponding to hair), with ‘white ball’ and ‘mushroom‐cap’ sign in Case 6 and thick white lines and bright white spots, with ‘cotton‐wool tufts’ in Case 7.

**Videoclip S12** Grayscale and power Doppler ultrasound imaging showing a multilocular cyst with different echogenicity of cyst fluid in different locules. Some cyst locules contain anechoic contents and others contain fluid with low‐level echogenicity. This cyst is described as having variable cyst contents (this was a histologically confirmed ovarian mucinous carcinoma, expansile type).

**Videoclip S13** Grayscale and power Doppler ultrasound imaging showing different types of shadowing. Case 1 shows fan‐shaped shadowing (histologically confirmed ovarian fibroma). Case 2 shows shadowing behind a ‘white ball’ in a histologically confirmed dermoid cyst. Case 3 shows shadowing behind calcifications in a papillary projection in a unilocular‐solid cyst (histologically confirmed serous cystadenofibroma). Case 4 manifests edge shadows from a unilocular cyst (functional cyst on follow‐up). Edge shadows are not classified as acoustic shadows.

**Videoclip S14** Grayscale ultrasound imaging showing ascites with fluid above the level of the uterine fundus. In Cases 1 and 2, transvaginal ultrasound demonstrates fluid above the level of the uterine fundus (Case 1: histologically confirmed tubo‐ovarian high‐grade serous carcinoma, FIGO Stage IVB; Case 2: histologically confirmed tubo‐ovarian high‐grade serous carcinoma, FIGO Stage IIIC). In Case 3 (same patient as in Case 2), on transabdominal ultrasound, fluid in Morrison's pouch is detected in sagittal (SAG) and transverse (TRANS) planes.

**Videoclip S15** Grayscale ultrasound imaging showing measurement of a multilocular cyst with anechoic contents. The length and anterior–posterior diameters of a lesion are measured perpendicular to each other in a sagittal section through the cyst (SAG) at the point at which the lesion appears to be at its largest. Then, the transducer is rotated 90°, to obtain a transverse section through the lesion, perpendicular to the sagittal plane, and the maximum transverse diameter of the lesion is measured. If the cyst capsule is visible, it is included in the measurement of the lesion.

**Videoclip S16** Grayscale ultrasound imaging showing measurement of a single papillary projection in a unilocular‐solid cyst. The height of the papillary projection and the first diameter of its base are measured in a sagittal section (SAG) through the cyst, in the plane in which the papillary projection appears largest. The thickness of the cyst wall from which the papillary projection arises is not included in the height measurement. Then, the transducer is rotated 90°, to obtain a transverse section (TRANS) through the lesion, perpendicular to the sagittal plane, in which the second, orthogonal diameter of the base of the papillary projection is measured, at its maximum dimension. If there is more than one papillary projection, the largest is measured.

**Videoclip S17** Grayscale ultrasound imaging showing assessment of number of papillary projections in a unilocular‐solid cyst with multiple papillary projections (histologically confirmed serous borderline ovarian tumor). How to distinguish a single papillary projection with multiple peaks from multiple separate papillary projections is illustrated: by calculating the x/y ratio for each adjacent pair of papillary peaks and the valley between them. Example 1, in upper part of lesion: for an adjacent pair of peaks, if ‘x’ (height of the bottom of the valley) divided by ‘y’ (height of the lower of the two peaks, measured from the same baseline) is ≥ 50%, the two peaks are counted as a single papillary projection (marked 1). Example 2, in lower part of lesion: if x/y is < 50%, the two adjacent peaks are counted as separate papillary projections; thus, three papillary projections are present in this example (marked 1–3).

**Videoclip S18** Grayscale and power Doppler ultrasound imaging showing assessment of number of cyst locules in a multilocular cyst with four cyst locules and low‐level echogenicity of cyst fluid (suggestive of mucinous cystadenoma).

**Videoclip S19** Grayscale and power Doppler ultrasound imaging showing a solid tumor with multiple internal cysts (high‐grade serous carcinoma). The tumor is defined as solid because on ultrasound it is estimated to consist of at least 80% solid tissue; the internal cysts are therefore not counted.

**Videoclip S20** Videoclip illustrating the effect on the ultrasound image of different Doppler ultrasound settings in the assessment of a multilocular‐solid cyst (high‐grade serous carcinoma at final diagnosis). Examples 1, 2 and 3 demonstrate the difference between power Doppler ultrasound images with fixed gain (Gn, 2.0) and pulse repetition frequency (PRF) decreasing from 0.9 to 0.6 to 0.3 kHz. Examples 4, 5 and 6 demonstrate the difference between power Doppler ultrasound images with a fixed PRF of 0.6 KHz and Gn decreasing from 8.4 to 4.4 to 3.0. Examples 7, 8 and 9 demonstrate the difference between power Doppler ultrasound images with a fixed PRF of 0.3 KHz and Gn decreasing from 7.4 to 5.0 to 3.0.

**Videoclip S21** Grayscale and power Doppler ultrasound imaging showing assessment of the color content of a tumor. Case 1 shows absence of blood in the tumor, i.e. color score of 1 (dermoid cyst). Case 2 shows minimal color content, i.e. color score of 2 (multilocular cyst suggestive of mucinous cystadenoma). Case 3 shows moderate color content, i.e. color score of 3 (ovarian fibroma with focal ovarian stromal hyperplasia). Case 4 (high‐grade serous carcinoma) and Case 5 (cystadenofibroma) show abundant color, i.e. color score of 4. In Case 5, a color score of 4 is given, despite the wall and septa being only moderately vascularized, because there is one highly vascularized solid component (‘worst‐case scenario’ rule).

**Videoclip S22** Grayscale and power Doppler ultrasound imaging showing, in a unilocular cyst, the cogwheel sign, caused by thickened endosalpingeal mucosal folds of the Fallopian tube (pelvic inflammatory disease).

**Videoclip S23** Grayscale and power Doppler ultrasound imaging showing the beads‐on‐a‐string sign (chronic hydrosalpinx). A cross‐sectional view of the Fallopian tube reveals tiny protrusions into a fluid‐filled dilated tube. These correspond to fibrotic, flattened endosalpingeal fold remnants.

## Data Availability

Data sharing not applicable to this article as no datasets were generated or analysed during the current study.
