# Supplementary figures and images for: Terms, definitions and measurements to describe the sonographic features of adnexal tumors: updated consensus opinion from the International Ovarian Tumor Analysis (IOTA) Group
Source: Ultrasound Obstet Gynecol. 2026 Apr 7;67(5):702–24. doi: 10.1002/uog.70191 (PMC13136064; doi:10.1002/uog.70191)

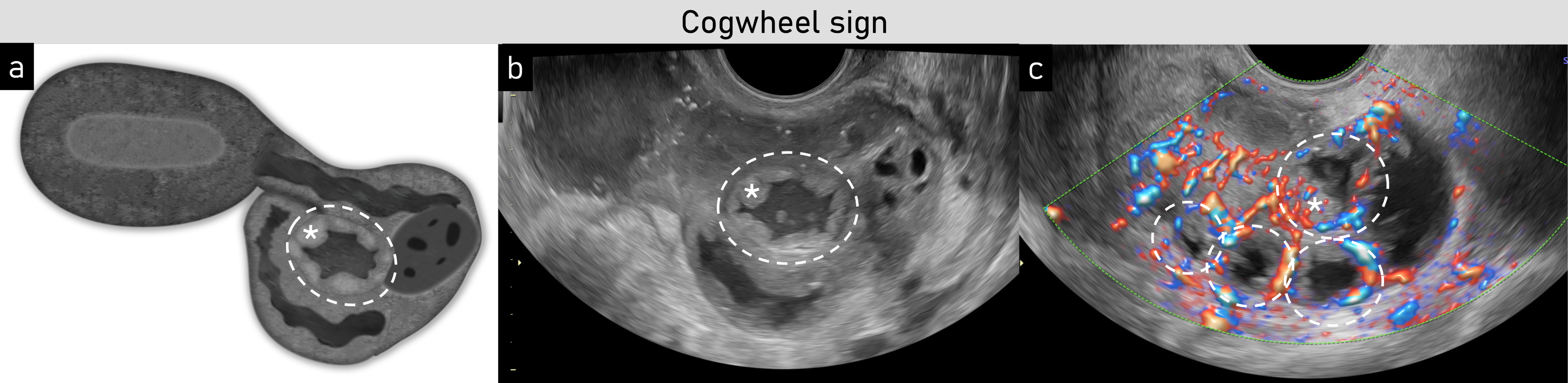

Supplement: Supplementary file 1 — Figure S1 Cogwheel sign. (a) Schematic drawing showing the cogwheel sign (encircled by dashed line), which is caused by swollen mucosal folds protruding into the lumen of a fluid‐filled, inflamed Fallopian tube. Grayscale (b) and power Doppler (c) ultrasound images of an inflamed Fallopian tube filled with fluid of low‐level echogenicity. In longitudinal section, incomplete septa (pseudosepta) are seen, corresponding to folding (‘kinking’/doubling‐up) of the distended tube, resulting in a unilocular cystic appearance. In cross‐section, thickened mucosal folds (*) produce the characteristic cogwheel appearance (encircled by dashed lines). See also Videoclip S22. [file UOG-67-702-s017.png]

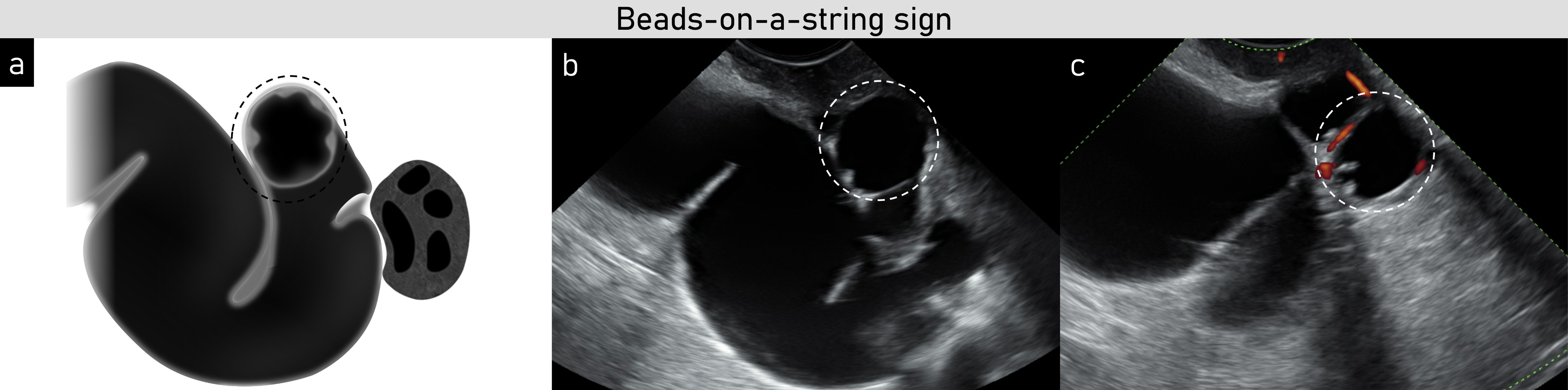

Supplement: Supplementary file 2 — Figure S2 Beads‐on‐a‐string sign. (a) Schematic drawing showing the beads‐on‐a‐string sign (encircled by dashed line) in the cross‐section of a Fallopian tube. Grayscale (b) and power Doppler (c) ultrasound images of a chronic hydrosalpinx, showing tiny protrusions into the fluid‐filled tube, with ‘beads‐on‐a‐string’ appearance in cross‐section (encircled by dashed line). The tiny protrusions correspond to fibrotic, flattened endosalpingeal folds. See also Videoclip S23. [file UOG-67-702-s012.png]
